# Morbillivirus and Pilot Whale Deaths, Mediterranean Sea

**DOI:** 10.3201/eid1405.070948

**Published:** 2008-05

**Authors:** Antonio Fernández, Fernando Esperón, Pedro Herraéz, Antonio Espinosa de los Monteros, Cristina Clavel, Antonio Bernabé, J. Manuel Sánchez-Vizcaino, Philippe Verborgh, Renaud DeStephanis, Francisco Toledano, Alejandro Bayón

**Affiliations:** * University of Las Palmas de Gran Canaria, Las Palmas de Gran Canaria, Canary Islands, Spain; † National Institute for Crop and Food Research, Madrid, Spain; ‡ University of Murcia, Murcia, Spain; § University Complutense-Madrid, Madrid, Spain; ¶ CIRCE (Conservation, Information, Research, Cetaceans) Andalusia, Spain; # PROMAR (Recovery Program for Marine Animals), Almería, Spain

**Keywords:** pilot whales, mortality, morbillivirus, dispatch

## Abstract

An outbreak of a lethal morbillivirus infection of long-finned pilot whales occurred in the Mediterranean Sea from the end of October 2006 through April 2007. Sequence analysis of a 426-bp conserved fragment of the morbillivirus phosphoprotein gene indicates that the virus is more closely related to dolphin morbillivirus than to pilot whale morbillivirus.

Morbilliviruses have emerged as serious pathogens of cetaceans and pinnipeds worldwide (*1*). The 2 cetacean morbilliviruses that have been identified are porpoise morbillivirus (PMV), isolated from harbor porpoises that died along the coast of Ireland, and dolphin morbillivirus (DMV), first identified in striped dolphins from the Mediterranean Sea (*1*,*2*). Although to our knowledge, morbillivirus outbreaks in pilot whales have not been previously reported, antibodies to morbilliviruses have been reported in 86% of 2 species of pilot whales (*Globicephala melas* and *G. macrorrhynchus*) in the western Atlantic (*3*). Barrett et al. found that 93% of stranded long-finned pilot whales (*G. melas*) were seropositive for morbillivirus, which provides more evidence that cetacean morbilliviruses are widespread (*4*). Molecular evidence from a pilot whale that was stranded on the coast of New Jersey, USA, and died from encephalitis, suggested that the long-finned pilot whale is host for a different, novel type of cetacean morbillivirus (pilot whale morbillivirus [PWMV]), which is distinct from PMV and DMV (*5*). We report an epizootic of lethal morbillivirus infection in long-finned pilot whales that occurred in the Mediterranean Sea.

## The Study

During a 6-month period (end of October 2006 through April 2007), >27 long-finned pilot whales were found stranded, 6 alive and 21 dead, along the southern Spanish Mediterranean coast and Balearic Islands. According to information from the Andalucia regional stranding network, CIRCE, (Conservation, Information, Research, Cetaceans), nongovernment organizations, and scientists working on that coastal area, 10 of these pilot whales were stranded in the Strait of Gibraltar area from the end of October 2006 through early February 2007. From January through April 2007, 7 of these whales were found stranded on the Almería coast, 6 on the Murcia coast, 2 on the Valencia coast; another 2 were found beached on the Baleares Islands. The Table compares the times and locations of these strandings with those of historical strandings.

Of these stranded whales, 18 were found in an advanced autolytic condition, but 9 were fresh or only moderately autolytic, of which complete necropsies were performed on 7, partial necropsies on 2, and samples were collected from all 9. Histologic and immunohistochemical examination of formalin-fixed tissues (mainly lymph node, brain, esophagus, liver, and kidney) was performed for 9 whales, and a virologic examination was performed on frozen tissues (mainly lymph node, lung, and brain) from 6.

According to biological and morphometric parameters, all stranded pilot whales were adults or subadults, except 2 that were juveniles. One female whale stranded off Baleares Islands was 7 months pregnant. For most of the stranded whales, the main macroscopic findings detected during the necrospsy were moderate to severe cachexia, represented by marked loss of volume of epaxial musculature. Stomachs were empty. In 3 whales, subcutaneous tissues were yellowish (icteric) and edematous. All necropsied whales had enlarged edematous lymph nodes, which showed parenchymal multifocal necrosis (especially digestive tract lymph nodes). Erosive stomatitis and erosive-to-ulcerative necrotizing esophagitis was detected in 3 whales. For 2 whales, the urinary bladder was empty and had thickened walls containing yellowish dense mucus in the lumen.

Microscopically, the main lesions were found in lymph nodes, which had a multifocal necrotizing lymphoadenitis and multinuclear syncytial cells. A nonpurulent encephalitis with syncitial cells and intranuclear inclusion bodies, intracytoplasmic inclusion bodies, or both, were detected in 6 whales from which neurologic tissues were analyzed microscopically. Mild interstitial pneumonia was detected in 4 whales, but inflammatory lesions of the lung were absent in the others. One whale, stranded in Murcia, had a focal pyogranulomatous pneumonia caused by *Aspergillus* sp. Mild to severe, erosive to ulcerative necrotizing esophagitis was detected microscopically in all analyzed whales found to have gross lesions in this organ.

**Figure 1:**
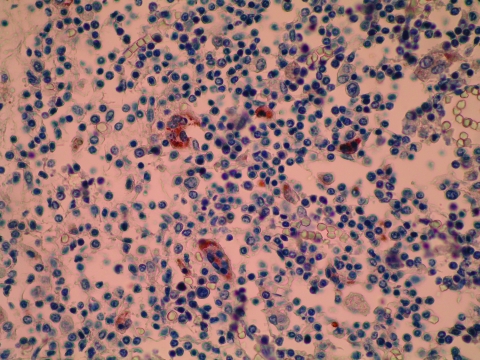
Lymph node of pilot whale. Positive intracytoplasmic immunoperoxidase staining of morbilliviral antigen in several syncytial cells and in monocyte-like cells. Avidin-biotin-peroxidase with Harris hematoxylin counterstain. Original magnification ×400.

Immunohistochemical staining, with a polyclonal antibody (*6*), showed morbillivirus antigen in bronchiolar epithelium, syncytial cells, monocyte-like cells, and cell debris of affected lymph nodes and brain; these tissues often showed a positive intracytoplasmic globular or granular immunoreaction. Morbillivirus antigen was detected in all whales for which an immunohistologic study was performed, mainly in the brain (n = 6), lymph nodes (n = 9), and lungs (n = 4) (Figure 1).

Reverse transcription–PCR (RT-PCR) to detect cetacean morbillivirus (CetMV) was performed for available samples of brain, lung, spleen, lymph node, liver, and kidney from 6 of the pilot whales and 1 fetus. Molecular detection of CetMV was performed by a 1-step RT-PCR of a 426-bp conserved region of the phosphoprotein gene, described previously (*7*). We conducted a BLAST (www.ncbi.nlm.nih.gov/blast/Blast.cgi) search to compare sequenced products with sequences described in the GenBank for morbillivirus. All sequences alignments were obtained, and p-distances were calculated by using MEGA version 3.1 (*8*).

**Figure 2:**
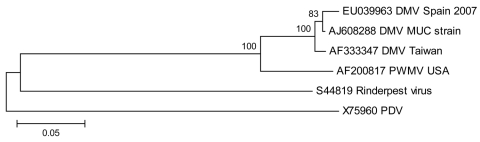
Neighbor-joining phylogram of 6 selected sequences from marine mammal morbilliviruses and Rinderpest virus. The name of the sequence indicates the GenBank accession number, virus species, and the country of the isolate. DMV, dolphin morbillivirus; PWMV, pilot whale morbillivirus; PDV, phocine distemper virus. The scale bar indicates the p-distance of the branches.

Of those whales analyzed for virus (6 pilot whales and 1 fetal whale), a morbillivirus was detected by RT-PCR in the brains of 5, lymph nodes of 6, and the lungs of 4. All samples from the fetus (brain, lung, lymph nodes, liver, and kidney) were RT-PCR positive for morbillivirus. Sequencing showed the same sequence in all positive samples from animals stranded in different areas of the southern coast of Spain (Figure 2). The novel sequence obtained was closely related to DMV (p-distance 0.01–0.03) and less closely related (more divergent) to PWMV (p-distance 0.11).

## Conclusions

The morbillivirus epizootic reported here induced high mortality rates among long-finned pilot whales in the Mediterranean Sea (Table). The epizootic had a spatiotemporal sequence, involving the long coast from southern Spain, beginning October–November 2006 in the Strait of Gibraltar, spreading eastward to Almería and finally northeast to Murcia; the last cases were detected in Valencia and the Balearic Islands in April 2007. High mortality rates among striped dolphins (*Stenella coeruleoalba*) have been noted since July 2007 in those coastal areas (currently under investigation along the coasts of Almería, Murcia, Valencia, and Catalunian) (data not shown). In our laboratories, a DMV has been isolated from 3 of those stranded striped dolphins (1 stranded along Murcia and 2 along the Almería coasts). This virus is molecularly almost identical to that reported here as affecting pilot whales (F. Esperón, pers. comm.).

**Table:** Pilot whale strandings, historical and epizootic, Mediterranean coastal area

Area, dates of historical records	No. stranded	Average no. strandings/y	No. historical strandings, 1998–2006 (dates)	No. epizootic strandings, 2006–2007 (dates)
Strait of Gibraltar, 1998–Sep 2006	8	0.9	3 (1998–2006 Oct –Feb)	10 (2006 Oct–2007 Feb)
Almería, 1998–Dec 2006	22	2.4	2 (1998–2006 Jan–Apr)	7 (2007 Jan–Apr)
Murcia, 2004–Dec 2006	12	4	1 (2004–2006 Jan–Apr)	6 (2007 Jan–Apr)
Baleares Islands, 1999–Dec 2006	2	0.25	Not known	2 (2007 Apr)
Total	44	7.55		25 (2006–2007 Oct–Apr)

The first morbillivirus epizootic described in cetaceans involved striped dolphins in the Mediterranean Sea in the 1990s when a DMV was described (*1*,*2*). Because the viruses isolated from those striped dolphins and these pilot whales are closely related phylogenetically, interspecies transmission should be considered. This epidemiologic point is reinforced by a new die-off event of striped dolphins in Mediterranean waters associated temporally and spatially with the pilot whale deaths caused by a DMV reported here. In the pilot whales the central nervous and lymphatic systems were the most severely affected tissues. Although pilot whales worldwide may be enzootically infected with morbillivirus (*9*), the virus involved in the present epizootic differs from PWMV (*5*), which supports previous evidence that different strains of CetMV may be infecting dolphins and whales (*10*).

Possible explanations for how and why the disease starts are, among others, pollutants (*11*), the high intensive chronic anthropogenic effects in the Strait of Gibraltar area, a DMV entering a naive pilot whale population, or a progressive decreasing of humoral immunity against the virus in these populations (*12*). Further research is needed to investigate the role of morbilliviruses on the health and massive deaths of pilot whales and other cetaceans.
